# O032. Associations between any headache and obesity: results from a systematic review and meta-analysis of observational studies

**DOI:** 10.1186/1129-2377-16-S1-A53

**Published:** 2015-09-28

**Authors:** Diana Degan, Raffaele Ornello, Cindy Tiseo, Amleto Gabriele, Francesca Pistoia, Antonio Carolei, Simona Sacco

**Affiliations:** Department of Neurology, University of L'Aquila, L'Aquila, Italy

## Background

Data about the association between migraine and obesity are controversial, but a recent meta-analysis showed an association between the two conditions[1]. As numerous studies addressing the possible association between any headache (including migraine) and obesity have provided conflicting findings[2, 3], we therefore performed a systematic review and meta-analysis of observational studies, to assess the associations between any headache and obesity and pre-obesity.

## Materials and methods

Data were obtained through multiple electronic databases up to April 2015, using the terms “migraine" OR “headache” in combination with “obesity" OR “pre-obesity" OR “body mass index”. Out of 2,022 records, we finally included 4 studies[4–7]. We obtained pooled adjusted effect estimates (PAEE) of the risk of having any headache in obese and pre-obese subjects versus normal weight subjects, and in obese and pre-obese women versus normal-weight women. To obtain PAEE the natural logarithm of each single estimate was weighed by the inverse of its variance. Only studies written in the English language reporting a clearly, unequivocal definition of exposure and outcome variables were included. To reduce methodological heterogeneity we performed our analyses including only the studies which defined BMI categories according to the World Health Organization (WHO) criteria for Western populations (underweight, <18.50 kg/m2; normal range, 18.50 - 24.99 kg/m2; overweight, ≥25.00 kg/m2; pre-obesity, 25.00 - 29.99 kg/m2; obesity, ≥30.00 kg/m2).

## Results

We found an increased risk of any headache in obese versus normal-weight subjects in 3 studies[5–7]; overall PAEE 1.29, (95% confidence interval [CI] 1.04-1.60; p = 0.022); in obese versus normal-weight women in 2 studies[4, 5]; overall PAEE 1.41, (95% CI 1.23-1.62; p < 0.001) and in pre-obese versus normal-weight women in 2 studies[4, 5]; overall PAEE 1.13, (95% CI 1.01-1.25; p = 0.025). When considering pre-obese versus normal-weight subjects in 3 studies[5–7], we did not find an increased risk of any headache (overall PAEE 1.06, 95% CI 0.94-1.18; p = 0.335).

## Conclusions

The meta-analysis of the available observational studies suggested an association between any headache and obesity, that was stronger in the female gender, and between any headache and pre-obesity in the female gender; these results are in line with the previous meta-analysis findings[1].
